# The Application of Artificial Intelligence in the Genetic Study of Alzheimer’s Disease

**DOI:** 10.14336/AD.2020.0312

**Published:** 2020-12-01

**Authors:** Rohan Mishra, Bin Li

**Affiliations:** ^1^Washington Institute for Health Sciences, Arlington, VA 22203, USA; ^2^Georgetown University Medical Center, Washington D.C. 20057, USA

**Keywords:** Alzheimer’s disease, genetics, artificial intelligence, machine learning

## Abstract

Alzheimer's disease (AD) is a neurodegenerative disease in which genetic factors contribute approximately 70% of etiological effects. Studies have found many significant genetic and environmental factors, but the pathogenesis of AD is still unclear. With the application of microarray and next-generation sequencing technologies, research using genetic data has shown explosive growth. In addition to conventional statistical methods for the processing of these data, artificial intelligence (AI) technology shows obvious advantages in analyzing such complex projects. This article first briefly reviews the application of AI technology in medicine and the current status of genetic research in AD. Then, a comprehensive review is focused on the application of AI in the genetic research of AD, including the diagnosis and prognosis of AD based on genetic data, the analysis of genetic variation, gene expression profile, gene-gene interaction in AD, and genetic analysis of AD based on a knowledge base. Although many studies have yielded some meaningful results, they are still in a preliminary stage. The main shortcomings include the limitations of the databases, failing to take advantage of AI to conduct a systematic biology analysis of multilevel databases, and lack of a theoretical framework for the analysis results. Finally, we outlook the direction of future development. It is crucial to develop high quality, comprehensive, large sample size, data sharing resources; a multi-level system biology AI analysis strategy is one of the development directions, and computational creativity may play a role in theory model building, verification, and designing new intervention protocols for AD.

## Introduction

Alzheimer's disease (AD) is a neurodegenerative disease characterized by progressive exacerbation of dementia; finally, patients lose the ability to respond to their environment. Except for cholinesterase inhibitors and memantine, which can alleviate or stabilize symptoms for a limited time, there is currently no way to stop the progression of AD [1]. With the aging of the world's population, AD not only causes more personal and family suffering, but caregivers of patients with AD have a higher prevalence of depression and anxiety, and AD becomes a serious social burden [2]. It is estimated that currently one in 10 Americans (10%) aged 65 and older has AD. In 2019, there were approximately 5.8 million AD patients in the United States. By 2050, this number may grow to a projected 13.8 million in the US, and the number of people living with dementia will reach 131.5 million worldwide [3-5]. Based on the age of onset, AD is classified as early-onset AD (EOAD) and late-onset AD (LOAD). EOAD accounts for approximately 5% of all AD cases and occurs in patients under 65 years old. Among them, less than half of the patients carry a causal mutation that manifests as an autosomal dominant inheritance pattern, known as early-onset familial AD. LOAD accounts for approximately 95% of all AD cases and occurs in patients over 65 years old. Based on the existence of family aggregation, AD can also be divided into familial and sporadic cases. Familial cases are mainly EOAD, but there are also cases of LOAD. More than 90% of AD patients are sporadic cases that are usually also LOAD [6]. The etiology for most cases of AD remains unclear and is thought to be the result of a complex interaction between genetic and environmental factors involved in neurobiological and immunological processes [7, 8], but an estimated 70% of the risk is attributable to genetic factors [9-12]. Currently, the amyloid hypothesis is a prevalent theory of the pathogenesis of AD. This hypothesis holds that a variety of factors cause imbalance in the production and clearance of β-amyloid, leading to the accumulation of β-amyloid in the brain, which in turn leads to neuroinflammation and the formation of neurofibrillary tangles in neurons, that will eventually lead to neuronal dysfunction and death, but the detailed pathological process is unclear [13].

One of the important goals of medical research is to discover the genetic and environmental factors that cause disease, namely, etiology studies, and the results can provide clues for researching the prevention and treatment of AD. Since the 17th century, Newton's methodology has been widely used in scientific research, including medical research, which emphasizes that the world’s apparent complexity can be solved by analyzing phenomena and simplifying them into the simplest components. In fact, we have achieved great success by utilizing this methodology. In the medical field, we have been able to successfully prevent and treat many diseases caused by one or several factors. For example, smallpox has been completely eliminated on Earth with the use of vaccines. However, currently, there are no solutions for the prevention and reversible treatment of certain complex diseases, such as AD, mainly because these diseases involve complex interactions between many factors, and the complexity of humans makes it impossible to use a simplified model to understand these diseases [14, 15]. In recent years, the rapid development of artificial intelligence (AI) technology has provided an opportunity to solve these problems involving massive data and ultracomplex structures that exceed the processing capabilities of the human brain [16-18]. In the field of AI, AD ranked fourth among all diseases in terms of the number of AI studies conducted [19]. AI adopts an integrative approach and model neurobiological components as functional modules of pathophysiology embedded within the complex, social dynamics that influence the phenomenology of neuropsychiatric disorders [20]. Because genetic factors are the main cause of most AD cases, they have been the focus of AD pathogenesis research. In recent years, with the widespread use of microarray and next-generation sequencing technologies, research using genetic data has shown explosive growth. This situation urgently requires the help of AI technology. Currently, genetic research on AD using AI technology is continuously increasing. Therefore, this article has conducted a comprehensive review of the research in this field and provides a perspective on the direction of future developments.

## Artificial Intelligence

The use of tools could be considered to be an “extension” of the human body's natural function. In the same way, computers can act as “extensions” of the human brain’s functionality. With the rapid development of computer power, the acquisition of large amounts of data, and the theory of computation, AI has or almost “will surpass human performance in several domains” [21, 22]. Intelligence is and has been humanity’s most useful ability for thriving on earth. There is reason to believe that with the development of AI technology, human productivity will enter a new era. AI is used in a broader scope with many definitions from different perspectives, and the ones that are commonly accepted include that AI is a branch of computer science that enables computers to perform tasks that generally require human intelligence; another perspective is that AI is a system that perceives the environment and takes action with a maximal possibility to complete a task [23]. To achieve “intelligent” functions, a large number of algorithms, methods, or strategies have been developed, and the main approaches of AI technology are problem solving by searching, knowledge-based reasoning and planning, uncertain knowledge-based reasoning, and learning from examples. The methods or strategies of problem solving by searching include uninformed or heuristic searches, local searches, optimizations, evolutionary computations, and adversarial searches. Knowledge-based reasoning and planning include logic programming, automated reasoning and ontological engineering. Uncertain knowledge-based reasoning includes Bayesian networks, hidden Markov models, Kalman filters, a utility theory, and decision networks. Learning from examples is based on a mathematical/statistical classification and machine learning. Among them, machine learning is the most widely used AI technique in academia and industry [24, 25].

Machine learning is a subset of AI that focuses on designing a computer program that automatically improves through experience. It works with datasets by examining the data and learning patterns within it. Most machine learning approaches fall into two categories: supervised and unsupervised learning algorithms. Supervised learning algorithms use labeled data, i.e., training data that have the correct result given an input, and these are best for classification and regression tasks. The common algorithms include artificial neural networks, Bayesian networks, support vector machines, decision trees, random forests, and K-nearest neighbors. In contrast, unsupervised learning algorithms use unlabeled data, where the algorithm needs to find and learn inherent patterns within the dataset. The common algorithms include K-means, distance clustering, density clustering, hierarchical clustering and Markov chain. There are also some algorithms that combine supervised and unsupervised learning, for example, reinforcement learning [24, 26, 27]. Deep learning is an even more specific subset of AI and machine learning. Deep learning is a machine learning algorithm that simulates a human brain approach to solving problems. It consists of several “layers” each with a various number of nodes all interconnected in a network. Information is input into the first “layer” and goes through several linear transformations until it outputs a result at the end. It can be supervised, unsupervised or enhanced, depending on how it is applied [28]. Before AI, many projects were implemented with complicated rule-based algorithms, which would continuously grow more complicated as more anomalies were discovered in the data. We can continuously make more rules and algorithms to try and account for every possible oddity, but this becomes laborious and overcomplicated. However, a machine learning implementation can simply learn these patterns. Furthermore, machine learning will be able to discover additional patterns of increased complexity or abstraction within the data. As the quantity, quality, and diversification of data increases, the effectiveness of a machine to learn patterns and logic in it becomes more efficient. This expansion of data is especially noticeable in medicine because of the new approaches to collect large amounts of biological data, such as genomic and other omics biology datasets [29]. Therefore, AI will have a great future in the application of healthcare, including the prevention, detection, diagnosis, and treatment of diseases, the management of health systems, and the development of medical research [30, 31].

## The Applications of AI in Medicine

Shortly after the official naming of AI at a Dartmouth College meeting in 1956, research into the application of AI in the medical field began. The main research topic at that time was the Medical Diagnostic Decision Support System (MDDS). For example, Warner et al. developed a pioneering MDDS system that can be used to diagnose congenital heart disease [32, 33]. There was a period of low amounts of AI research from 1974 to 1993; however, due to the remarkable progress of computers and information technology in recent years, research on the application of AI in the medical field has become a hot topic [24]. Many information technology companies and research institutions are currently working on the development of AI technology in clinical medicine, health systems management, public health, and medical research. For clinical medicine, the development of computer vision, computer analysis of images and videos, and the combination of these with AI technology have significantly improved the classification and detection of images, and this is very beneficial for medical imaging. These technologies have been developed for imaging diagnosis in radiology [34], pathology [35], dermatology [36], ophthalmology [37], cardiology [38], neurology [39], gastroenterology [40], and surgery [41] have shown satisfactory results in many aspects. In addition, AI can predict the progress of disease and treatment effects by learning the health trajectory from a large number of patients. For instance, by using ^18^F-fluorodeoxyglucose PET of the brain, a deep learning algorithm for the early prediction of AD was developed, and this achieved 82% specificity and 100% sensitivity at an average of 75.8 months prior to the final diagnosis [42]. Therefore, it is believed that the use of AI technology in clinical facilities may help improve the quality of medical practice, which is particularly helpful for physicians with insufficient training or experience, especially in developing countries with insufficient medical resources [43]. AI technology can also expand the reach of medical services; for example, patients can use a smartphone or smart watch application for self-care, and some applications are currently approved by the FDA. Precision medicine is based on the patient's genetics, environment, and lifestyle factors and is tailored to his or her personal healthcare plan and the clinical decisions for the patient. AI technology can analyze and process very large amounts of genetics, environment and lifestyle data, and this allows for the ability of precision medicine to be applied in clinical practice. In addition, it may play an important role in health system management and public health [17, 27, 30, 44-46].

In the field of biomedical research, currently developed biological and medical technologies can extract a large amount of laboratory and clinical research data from complex biological processes, such as genomes, transcriptomes, proteomics, cytological images, chemical and biological macromolecular structures, interaction information, and clinical data from electronic medical records. AI technology can analyze and process large and complex biological data to help clarify the corresponding physiological and pathological mechanisms and then assist in designing and screening drug molecules and in designing and analyzing clinical trials. For research at the gene level, AI can help to predict the binding affinities of transcription factors, DNA- and RNA-binding proteins, cis-regulatory/enhancer elements, DNA methylation sites, histone modifications, chromatin accessibility, transcription start sites, tissue-regulated splicing, special gene expression and translation efficacies, transcriptome patterns in a particular cell or condition, microRNA precursors and binding targets, variant calling, functional consequences of noncoding variants, and coding variants pathogenicity. AI can also help to identify long noncoding RNAs, generate protein-coding DNA sequences, and design DNA probes for protein binding microarrays. As the amount of genomic data grows exponentially, deep learning seems to be the best way to analyze these data sources and complete genomic modeling tasks; however, the prediction of complex human disease phenotypes is currently far from mature [47-50]. For protein level research, current AI technologies can predict the secondary structure, solvent-accessible surface area, solvent accessible surface area, protein contact maps, and disordered regions; however, the prediction of the tertiary protein structure is still challenging [51, 52]. For cell- and tissue-level research, automated high-content, high-throughput imaging technology is not only a powerful tool for studying biomedical issues but also a tool used for all stages for developing target-based drugs. Specific functions of AI in image processing include signal denoising and enhancement, segmentation, label-less imaging, live cell imaging, imaging-based phenotype, single cell tracking, and modeling of reconstructed pedigree trees [53, 54]. With the application of AI technology in the chip laboratory, cell-based or organoid-based assays, and automatic compound synthesis, it is possible to greatly accelerate the development of new drugs. AI can be used to analyze literature and high-throughput compound screening data and to propose plans for initial molecular screening and automated chemical synthesis. After obtaining bioassay data, by updating the machine learning model, a new molecular optimization plan can be proposed, and the bioassay can be performed again. In this way, an automated drug development cycle based on AI design and high-throughput bioassay is formed [55]. Drug repurposing is a low-cost, rapid drug development pathway. AI technology can predict drug repurposing by analyzing large-scale transcriptomics, molecular structure data, and clinical databases [56]. Clinical trials are the bottleneck of new drug development, and researchers believe that the use of AI technology in the design and implementation of clinical trials can help solve this problem. By analyzing patient genetic and clinical data, AI can help select a subset of the population who may be sensitive to new drugs, and it can also help recruit subjects by matching patients and clinical trials. Combining AI technology with wearable sensors and noninvasive diagnostics during clinical trials can provide a mobile, real-time patient monitoring system and may predict the dropout risk of a particular patient [57]. Although research on AI-based medical technology has developed rapidly and has broad application prospects, there are few examples of clinical applications. It can be said that AI-based medical technology is still in its infancy [30, 58].

## Overview of Genetics Research on AD

It is estimated that for AD cases other than early-onset familial AD, genetic factors may contribute at approximately 70% of the etiologic role [9, 10]. The genetic variations between individuals in the population include single nucleotide variations (SNVs), tandem repeat variations, small insertions and deletions, large segment deletions and duplications (copy number variations), chromosome rearrangements (duplication, deletion, inversion, and translocation), and aneuploidy or polyploidy (often causing major genetic diseases) [59]. The human genome is approximately 3.2 × 10^9^ base pair (bp), of which the noncoding region accounts for approximately 99% of the entire human genome. The noncoding region also has important cellular regulatory functions and includes regulatory elements (promoters, enhancers, silencers, and insulators); production of transfer RNA, ribosomal RNA, microRNAs; long noncoding RNAs; formation of structural elements of the chromosome, including telomeres, satellite DNA, etc. [50, 60, 61]. To discover genetic variations associated with the development of AD in the human genome, four strategies have been applied: genetic linkage analyses, candidate gene/pathway association studies, genome-wide association studies (GWAS), and next-generation sequencing (NGS)-based association studies [62].

Genetic linkage analysis is one of the earliest strategies for assessing the genetic basis of Mendelian traits. It maps genetic loci through genetic markers and segregation analysis in pedigrees [63]. Through genetic linkage analysis, causal mutations in three genes, amyloid precursor protein (*APP*), presenilin 1 (*PSEN1*), and presenilin 2 (*PSEN2*) were found in early-onset familial AD [57]. They are located on chromosomes 21, 14, and 1, respectively [64]. Individuals with Down syndrome carry an extra copy of chromosome 21, which is responsible for the pathological changes of EOAD [65].

Candidate gene/pathway approaches are small-scale and low-resolution association studies based on existing knowledge of some genes. This approach has identified the apolipoprotein E gene (*APOE*) alleles as risk factors for late-onset AD. Although this method is not commonly used today, it is still valuable depending on the gene or population, for example, when exploring polymorphisms with low allele frequencies [66, 67].

Based on advances in microarray technology, GWAS can assess the association of thousands of single nucleotide polymorphisms (SNPs) of a disease and provide information on genetic variations associated with the risk of certain diseases [68]. Certain international cooperation projects, such as the International Alzheimer's Disease Project (IGAP), have conducted large GWAS samples of LOADs involving tens of thousands of patients [69, 70].

GWAS has confirmed that *APOE ε4* is the most important genetic risk factor for AD, but more susceptible loci have been reported recently and include *ABCA7, ACE, ADAM10, ADAMTS1, ATP5H-KCTD2, BIN1, BZRAP1, CASS4, CD2AP, CD33, CELF1, CLU, COBL, CR1, DSG2, EPHA1, FBXL7, FERMT2, FRMD4A, HBEG F, HLA-DRB5-HLA-DRB1, INPP5D, IQCK, MEF2C, MS4A4E/MS4A6A, MTHFD1L, NME8, PFDN1-HBEGF, PICALM, PTK2B, RANBP2, SCIMP, SLC10A2, SLC24A4-RIN3, SORL1, SPPL2A, TREM2, TRIP4, USP6NL-ECHDC3, WWOX* and *ZCWPW1* [11, 62, 71]. The GWAS data are available from the National Human Genome Research Institute - European Bioinformatics Institute (NHGRI-EBI) GWAS catalogue (www.ebi.ac.uk/gwas/).

NGS uses revolutionary massively parallel sequencing technology that allows each base in the entire human genome to be quickly sequenced multiple times to provide comprehensive and accurate DNA data. Sequencing of all protein coding regions is called whole-exome sequencing (WES), and sequencing of the entire genome is called whole-genome sequencing (WGS) [72]. Since GWAS requires a large number of samples to achieve statistical significance, it is difficult to find extremely rare variants that affect the risk of AD, but NGS can obtain subjects’ complete genomic sequence data; therefore, it is possible to capture all such variants. NGS has led to the discovery of a few mutant genes in some cases with unexplained EOAD, and includes *NOTCH3*, *SORL1*, *TREM2*, etc. [73-75]. In some very small population groups, NGS has also discovered a number of susceptibility loci related to the development of AD, but GWAS usually missed them, such as *ARSA, CHMP2B, CSF1R, FSIP2, GRN, IGHG3, NCSTN, NOS1AP, PLD3, TM2D3, TTC3, ZBTB4, and ZNF655* [76-82].

With the exception of *APOE*, all of the genetic variations described above have little effect on the pathogenesis of AD. To understand the etiology of AD other than early-onset familial AD, it might be necessary to consider the effect of multiple variants (additive effects), epistasis (multiplicative effects), and the interaction of genes with the environment. Genetic risk scores can be used to describe the synthetic effects of multiple variants on the pathogenesis of AD by calculating the number of disease-related alleles and their power to predict the risk of AD. A genetic risk score study based on an SNP dataset including 3,049 AD cases and 1,554 controls showed that the maximum predictive accuracy for AD was 82% [83]. In epistasis studies, interactions were found in some genes that have never been associated with AD before, while the individual gene analyses did not show any effect, for example, the interacting SNP pair in *KHDRBS2* and *CRYL1* [84]. The results support that the epistasis effect also contributes to a portion of the heredity of AD. A review article by Raghavan and Tosto summarizes studies of gene-to-gene interactions in AD [85]. The concept of functional genomics attempts to link omics data such as genomics, transcriptomics, proteomics, and metabolomics to explain the complex process from genotypes to phenotypes at a comprehensive level. Functional pathway analysis showed that AD-related genetic variants were mainly enriched in APP metabolism, inflammation, lipid metabolism, tau protein binding, endocytic/vesicular-mediated transport and synaptic function pathways [11, 62, 70, 71, 86].

Many environmental factors have been found to increase the risk of AD, including brain trauma, low education levels, risk factors for cardiovascular disease, lifestyle (e.g., tobacco, alcohol consumption, exposure to greenery, and exercise) [87], air pollution [88], heavy metal exposure (e.g., mercury, manganese) [89, 90], pesticide exposure, etc. However, there is no evidence showing that these environmental risk factors alone are the cause of AD; it is speculated that they may trigger the pathogenesis of AD by interacting with the risk genes of an individual. Studies have evaluated the role of *APOE* genes in interacting with the environment. For example, if individuals have both the *APOE ε4* allele and low physical activity, their risk of developing dementia is much higher than those having only one factor [91]. However, there is very little research data on the interaction between genetic variations and environmental risk factors [92].

Epigenetics focus on mechanisms that affect gene expression without altering DNA sequences, including DNA methylation, histone modifications, chromatin remodeling and noncoding RNA regulation. Epigenetic modifications are influenced by external and internal environmental factors, such as age, lifestyle, disease status, pollution [93]. Many studies have reported epigenetic changes in the brains of AD patients, for example, changes in DNA methylation levels in *COASY, SPINT, BRCA1*, and *PLD3* promoter regions, *APOE* CpG islands and *HOXA* gene clusters, an overall reduction in DNA methylation in the cortex and hippocampus, increased histone deacetylase (HDAC) 6, decreased class III HDACs, changes in miRNAs (miR-29 family, miRNA-7, miRNA-9-1, miRNA-23a/miRNA-27a, miRNA-34a, miRNA-125b-1, miRNA- 146a, and miRNA-155) and long noncoding RNA (BC200 RNA) levels in the AD brain. These findings have been summarized in some reviews [62, 94].

It has been reported that some mitochondrial haplogroups and single nucleotide polymorphisms affect the risk of AD, but due to the small sample size and lack of validation, the contribution of mitochondrial genetic variation to AD risk is inconclusive [95, 96].

As mentioned above, most cases of AD are thought to be caused by complex interactions between multiple genetic variations and environmental risk factors. High-quality large-scale databases and powerful bioinformatics methods may be the main ways to help understand this complex interaction, specifically the global-scale AD research shared database and AI technology. Some important data sharing resources in AD research are: Alzheimer’s Disease Genetics Consortium (ADGC, www.adgenetics.org), Alzheimer’s Disease Sequencing Project (ADSP, www.niagads.org/adsp/content/home), Alzheimer’s Disease Neuroimaging Initiative (ADNI, http://adni.loni.usc.edu/), AlzGene (www.alzgene.org), Dementias Platform UK (DPUK, https://portal.dementiasplatform.uk/), Genetics of Alzheimer’s Disease Data Storage Site (NIAGADS, www.niagads.org/), Global Alzheimer’s Association Interactive Network (GAAIN, www.gaain.org/), and National Centralized Repository for Alzheimer’s Disease and Related Dementias (NCRAD, https://ncrad.iu.edu) [97, 98].

## The Applications of AI in AD Genetic Analysis

AI technology, mainly machine learning algorithms, has shown to be efficient for large data analysis of high-dimensional complex systems. Currently, machine learning has been used in the studies of diagnosis and prognosis of AD based on genetic data, the analysis of genetic variations, gene expression profiles, gene-gene interactions in AD, and genetic analyses of AD based on a knowledge base.

## Diagnosis and Prognosis

In earlier times, AI algorithms were used to make predictions for the diagnosis and prognosis of AD by analyzing patient genetic information. Takasaki et al. published two papers in 2008 and 2009 that studied centenarians and other patients in Japan with AD and Parkinson's disease.

In the first paper, they used a radial basis function (RBF) network to analyze mitochondrial single nucleotide polymorphisms (mtSNPs) at specific locations in mitochondrial DNA and found that different types of subjects have unique mtSNPs. Japanese patients with AD are closely related to the G2a haplogroup. The second paper showed that in addition to the G2a haplogroup, Japanese AD patients were associated with B4c1 and N9b1 haplogroups. The authors believe that this analysis method can be used for the preliminary diagnosis to predict the possibility of someone developing AD or several other diseases [99, 100]. In 2011, Wei et al. developed a model-averaged naïve Bayes (MANB) model that performs better than previous models in predicting LOAD patients with 312 to 318 SNPs in 1,411 patients. The area under the receiver operating characteristic curve (AUC) reached 0.72. In addition, the model performs better when trained and tested with high-dimensional genomic data. The results support that MANB can be used to predict AD from genome-wide data [101].

In a recent study, Xu et al. created a support vector machine (SVM) algorithm to analyze gene-encoded protein sequences instead of patient genotype data. The algorithm was tested with 279 AD-related protein sequence data and 1,463 non-AD-related data from the UniProt database, and the prediction accuracy reached 85.7%. However, the weakness of this study is that it does not distinguish protein sequence information between early-onset familial AD and other types of AD [102]. Wang et al. also used the SVM classifier to analyze the microarray gene expression dataset from the NCBI GEO database (www.ncbi.nlm.nih.gov/geo) to generate a gene coexpression network to identify possible AD diagnostic biomarkers. They identified a cluster of 44 genes as potential biomarkers [103].

Varatharajah et al. developed a multivariate model based on machine learning algorithms (SVM, multiple kernel learning), which integrates demographics, biomarkers of cerebrospinal fluid (CSF), magnetic resonance imaging (MRI), positron emission tomography (PET), a psychological test score for cognition and cognitive resilience, and top AD-related genes that have been validated (including a total of 94 potential predictive factors) to identify patients with mild cognitive impairment (MCI) who will progress to AD within three years. By analyzing 135 participants from the ADNI, their prediction accuracy rate was an astonishing 93% [104]. The above research shows that the analysis of genetic data based on machine learning methods to predict the prognosis and risk stratification of AD has certain value, but if combined with imaging data, its accuracy will be greatly improved.

## Analysis of genetic variations in AD

Since the use of GWAS to explore the genetic variation of AD, very high-dimensional SNPs have been generated, and most of these SNPs are irrelevant to AD. A study published by Wu et al. in 2012 proposed an effective stratified sampling method using a decision tree and Breiman random forest generation method to classify SNPs into multiple groups from an AD case-control dataset containing 380,157 SNPs [105]. Nguyen et al. also recognized the value of the random forest algorithm for identifying genetic variation from the GWAS dataset. An improved random forest method with quality-based two-stage sampling was tested on a Parkinson and an AD GWAS SNP dataset; the results showed that this method was effective in selecting subgroups of SNPs, and the performance was even better than traditional ones [106].

Hamed et al. applied SMV classifiers with different kernels to analyze the ADNI-1 dataset, and the results confirmed that the *APOE, ABCA7, BIN1, CD2AP, CD33, CLU, CR1, MS4A6A*, and *PICALM* loci found in previous GWAS were highly correlated with AD [107]. To address the challenge of individual data privacy concerns in the collaborative studies of GWAS from different institutions, Li et al. developed a processing framework that performs a sparse learning method (lasso regression analysis) in a distributed manner. Their method could exclude irrelevant features and rank SNPs that affect AD through risk without any privacy issues. An empirical study of SNP datasets from three independent institutions identified some risk SNP-associated genes for the diagnosis of AD, including *APOE*, *CD69*, and *PHACTR3* [108]. To improve the accuracy of identifying AD risk variants in the GWAS dataset, Lancour et al. suggested incorporating previously known information about AD candidate genes into the analysis. They developed an SVM approach that integrated genetic and network scores by propagating GWAS risk scores in a protein-protein interaction network to prioritize specific candidate disease genes from the ADGC GWAS dataset. A number of new AD risk candidate genes were predicted using this combination method. The top ten genes included *CR2, SHARPIN, PTPN2, C4B, TUBB2B, EPS8, PSMC3, STRAS, HSPA2*, and *STUB1*. Pathway analysis showed that the ranking genes based on this combination approach were significantly enriched in AD-related pathways, including immune response, aging and hippocampal development [109].

With the development of brain imaging technology, it has been found that certain changes in the brain structure and function can occur for years before the clinical diagnosis of AD [110]. Many researchers have analyzed the association between brain imaging changes and genetic variations, and this is called neuroimaging genetics. Since 2004, the ADNI project funded by the United States National Institutes of Health and pharmaceutical companies has tracked and collected clinical, imaging, genetic, and biochemical biomarker data for AD patients, and it has become one of the most important AD brain imaging data sharing resources.

Wang et al. conducted a study in 2012 that used the sparse multimodal multitasking learning method to analyze imaging and GWAS genetic data from the ADNI database to identify AD-sensitive biomarkers. They were able to predict disease status and identify a range of phenotypes and genetic biomarkers, the latter included *APOE, DAPK1, ENTPD7, SORCS1, BIN1, PICALM, SORL1, LOC651924, PRNP*, and *IL1B* [111]. Another group used a parallel version of the random forest algorithm to produce an AD risk gene ranking by associating GWAS data with multiple quantitative neuroimaging traits from the ADNI database. The top 10 genes within 10k bases of the top-risk SNPs include *TOMM40, APOE, PICALM, PVRL2, NTNG2, NTM, SLC12A1, MEF2D, CD109, UNC5B*, and *DPYD* [112].

Sparse canonical correlation analysis (SCCA) can be used to identify the multivariate associations between multiple SNPs and neuroimaging traits. Du et al. created two structural SCCA models to analyze the associations between genetic markers within the *APOE* gene and magnetic resonance imaging (MRI) and amyloid imaging data retrieved from the ADNI database. They found that the *APOE ε4* allele rs429358 was strongly associated with damage to the right hippocampus and amyloid burden in the frontal region [113, 114]. Hou et al. used a multitask learning model to perform regression analysis on SNP and MRI datasets of ADNI, and they found several risk genetic variants of AD related to *APOE, BCR, NPC2*, and *RFTN1* genes [115].

Certain genes may play a tissue-specific role in the pathogenesis of AD. The network-wide association study (NetWAS) method can apply machine learning algorithms to tissue-specific functional interaction networks to prioritize analyzing the results of GWAS. Song et al. used this method to analyze the ADNI GWAS dataset with the hippocampal volume as the phenotype and found that the protocadherin alpha gene cluster (*PCDHA*) may be a suspicious gene [116]. The above studies have studied the association between genetic variations and static neuroimaging phenotypes at a single time point without considering the dynamics of phenotypic changes. Hao et al. hypothesized that these changing phenotypes could explain the dynamic neurodegeneration process and proposed a “temporally constrained group sparse canonical correlation analysis framework” that was trained with time series data from the ADNI database. They also focused on SNPs near the APOE gene and found that this model could detect stronger associations than previous SCCA models, confirming that the loci rs76692773 and rs2075649 were top ranking; however, the longitudinal method questioned the impact of the risk locus rs429358 on the deterioration of AD [117].

**Table 1:** Genetic risk factors for AD revealed by AI analysis exclusively

Genetic risk factors for AD	Biological processes [https://www.genecards.org/]
*ANKRD36C* [118]	Ion channel inhibitor activity
*BCR* [115]	Protein tyrosine kinase activity, macrophage functions,
*C4B* [109]	Inflammation
*CACNA1C* [118]	Voltage-dependent calcium channel
*CD109* [112]	Inflammation
*CD69* [108]	Inflammation
*CLCN3* [118]	Voltage-gated chloride channel
*CR2* [109]	Inflammation
*DAPK1* [111]	Apoptosis modulation and signaling
*DHRSX* [118]	Autophagy
*DPYD* [112]	Uracil and thymidine catabolism.
*ENTPD7* [111]	Oxidative stress and DNA damage
*EPS8* [109]	Regulator of axonal filopodia formation in neurons
*FGF14* [118]	Nervous system development
*GALNT18* [118]	O-linked oligosaccharide biosynthesis
*GFRA1* [118]	Glial cell line-derived neurotrophic factor receptor family
*HSPA2* [109]	Molecular chaperone implicated in a wide variety of cellular processes
*IL1B* [111]	Inflammation
*ITGBL1* [118]	EGF-like protein family
*LOC101928478* [118]	Long intergenic non-protein coding RNA
*LOC442028* [118]	Long intergenic non-protein coding RNA
*MAF1* [118]	Repression of RNA polymerase III-mediated transcription in response to changing nutritional, environmental and cellular stress conditions
*MEF2D* [112]	Cell growth, survival and apoptosis
*NIPA1* [118]	Magnesium transporter that may play a role in nervous system development and maintenance.
*NPC2* [115]	Transport of cholesterol
*NTM* [112]	Neural cell adhesion molecule
*NTNG2* [112]	Neurite outgrowth of both axons and dendrites
*OR11H4* [118]	Odorant receptor
*PCDHA* [116]	Cell surface proteins of neurons and synaptic junctions
*PHACTR3* [108]	Nuclear scaffold in proliferating cells
*PPA1* [118]	Respiratory electron transport and ATP synthesis
*PRNP* [111]	Neuronal development and synaptic plasticity
*PTPN2* [109]	Cell growth, differentiation and mitotic cycle
*PVRL2* [112]	Cell junction organization and adherens junction
*RFTN1* [115]	Inflammation
*RIMS1* [118]	Synaptic vesicle exocytosis
*SHARPIN* [109]	Inflammation
*SLC12A1* [112]	Sodium-potassium-chloride cotransporter
*SORCS1* [111]	Neuropeptide receptor activity
*SORCS2* [118]	Receptor for the precursor forms of NGF and BDNF
*STRAP* [109]	kinase activity
*STUB1* [109]	Inflammation
*TUBB2B* [109]	Isoform of tubulin
*UNC5B* [112]	Axon guidance

WGS data provide a new impetus for revealing extremely rare mutations affecting AD risks that GWAS cannot determine. Yang et al. explored a WGS database retrieved from ADNI through the Lasso regression, which included 6 million valid SNPs, baseline volumes of entorhinal cortex and hippocampus and their volume changes within 24 months. The top genes associated with the risk SNPs for the baseline volume of entorhinal cortex and hippocampus were *APOE, ANKRD36C, GALNT18, GPC6, LOC442028, MAF1, OR11H4, PPA1*, and *RIMS1*. The top genes associated with the risk SNPs for the volume changes of EC and Hip were *BACE2, CACNA1C, CLCN3, DHRSX, FGF14, GFRA1, ITGBL1, LOC101928478, NIPA1, SORCS2*, and *VAT1L*. Many of them had never been reported and required further validation. There were also many SNPs whose associated genes could not be identified [118]. Yang et al. further expanded their research by using a novel two-level structured sparse regression model that introduced sparsities in both nucleotide-level and gene networks. The analysis of WGS SNP and neural image data from ADNI showed that this method could effectively predict the risk SNVs associated with AD risk genes [119]. From the results of the above 14 papers, we understand that using machine learning algorithms to analyze the SNP data of GWAS and WGS can detect new genes and SNVs that may be related to AD risk (Table 1).

## Analysis of the Gene Expression Profile in AD

Genetic variations alone or in combination with environmental factors can alter gene expression profiles in brain cells, cause abnormalities in the metabolism of certain proteins, and ultimately lead to pathological changes in AD. Studying changes in gene expression levels in cells of the brain is helpful to discover key genes and pathways related to the pathogenesis of AD, which may be targets for therapeutic intervention. High-throughput microarray and RNA-sequencing (RNA-Seq) based on next-generation sequencing technology can create a detailed view of the transcriptome of cell or tissue samples. Due to the high dimensionality and complexity of the data, they are hindered from gaining significant information about the biological processes of a specific disease. As a result, many studies have shifted from traditional statistical methods to machine learning methods for data analysis, effectively revealing complex biological characteristics.

In 2011, Kong et al. developed two unsupervised machine learning algorithms (independent component analysis, ICA, and nonnegative matrix factorization, NMF) to analyze the microarray dataset of the hippocampal gene expression of control and AD samples. They found that changes in expression levels of many genes in the hippocampus of AD patients were related to metal metabolism and inflammation [120]. Scheubert was able to find relevant genes by using a wrapper approach of genetic algorithm and support vector machine (GA/SVM), which performed more efficiently by finding sets of genes that are less repetitive and more significantly attributed to AD. Through analyzing a dataset consisting of six different brain regions from 87 AD patients and 74 healthy control samples, they identified some new candidate biomarkers for AD, including *LOC642711, PRKXP1, LOC283345, SST* and *LY6H* [121]. Panigrahi et al. applied an integrative systems biology approach to identify candidate genes and important biological processes among AD and aging. Supervised learning software and a self-organizing map implemented with an unsupervised artificial neural network were used to analyze three separate microarray datasets, and these included the CA1 region of the hippocampus, frontal lobe and blood mononuclear cells from AD and aging patients. Ten major classes of transcription factors and unique miRNA targets were identified as regulatory processes for AD in this study [122]. As the number of available microarray databases increases, many studies strive to find more significant genes using different methods or algorithms, such as the random forest method used by Nishiwaki et al. and the two-stage classifier consisting of relevance vector machine (RVM), SVM, random forest and extreme learning machine (ELM) classifiers developed by Miao et al. These methods both identified some candidate genes related to AD [123, 124].

Li et al. studied the relationship of gene expression changes in blood and brain tissues by analyzing four blood and one brain tissue gene expression dataset. They found that more than 77% of genes have the same regulatory direction in different tissues and disease states. SVM, random forest and logistic ridge regression (RR) models showed that mitochondrial dysfunction, the NF-kappa B signaling pathway and iNOS signaling were important dysregulation pathways in the pathogenesis of AD [125]. It is currently known that controlling transcription through microRNA molecules is a key process in the development of late-onset AD. Armananzas et al. proposed a new method to integrate gene expression data and sequence predictions with a machine learning method. They analyzed two microRNAs and two gene expression datasets in temporal lobe samples and found some previously unreported the regulation of AD-related microRNAs, including miR-106a, miR-504, and miR-142-3p [126].

Recent studies began using a more complicated approach, and many of these researchers believe that unconventional and complicated algorithms should be used to find any additional genes that play a role in AD. Martinez-Ballesteros et al. combined decision tree classifiers, quantitative rules and hierarchical clustering methods and completed training on multiple carefully prepared gene expression datasets. However, they also considered additional sources, such as a repository of already relevant AD genes, gene ontology, and a literature review or expert knowledge, to validate their results. They found that the expression of 90 genes in patients with AD were significantly different from that of controls [127].

RNA-Seq uses next-generation sequencing technology to check the presence and quantity of all RNA in a sample, including alternative gene-splicing transcripts, posttranscriptional modifications, gene fusions, mutations, miRNA, tRNA, and ribosome profiles. In comparison, the weaknesses of hybrid-based microarrays are that they can only detect predesigned sequences and that the quantitative range is relatively narrow [128, 129]. Mukherjee et al. proposed an iterative multiview classifier using the logistic regression method. They used this classifier to analyze an RNA-seq dataset of 2,114 samples from seven different brain regions of 1,100 patients to identify potential AD risk (driver) genes. They found that the highest ranked genes contained several genes closely related to AD, consistent with previous reports and that the results of the RNA-Seq data could be well verified by the GWAS data. Enrichment analysis found that in addition to well-known processes (such as immune response and amyloid processing), there are other processes (such as endocytosis, scavenger receptor activity and peptidase activity) that could lead to a new understanding of the mechanism of AD development [130]. Luo et al. conducted a small sample study using logistic regression classifiers by combining RNA-Seq data, a database from Online Mendelian Inheritance in Man (OMIM, www.omim.org/), and protein-protein interaction networks. The RNA-Seq dataset contains 9 AD subjects and 8 control subjects (GSE53697). They found that candidate AD genes were enriched in seven AD-related pathways, which included the NOD-like receptor signaling pathway, neurotrophin signaling pathway, and GnRH signaling pathway [131]. In summary, the 10 studies presented in this section show that the use of machine learning to analyze transcriptomes generated by microarrays and RNA-Seq can help discover genes and pathways that play important roles in the pathogenesis of AD.

## Gene-gene Interaction in AD

As mentioned above, gene-gene interactions have significant roles in the pathogenesis of AD. Machine learning algorithms have been used in studies of SNP epistatic interactions, transcript interaction networks and metabolic pathways. In 2011, Jiang et al. created a combinatorial epistasis learning method with a Bayesian network. They evaluated the performance of this method with different parameters on simulated datasets and a real Alzheimer’s GWAS dataset, and the results showed that this method is feasible [132]. Later, Jiang et al. combined Bayesian network and information gain algorithms to further improve the method. They analyzed a GWAS LOAD dataset that included 859 AD and 552 control cases. The results not only were consistent with previous reports but also indicated new interactions, i.e., APOE / GAB2 interactions involving more loci [133]. Han et al. also used a Bayesian network-based method to detect epistatic interactions from the same GWAS LOAD dataset as Jiang et al. They found two SNPs (rs1931565 and rs4505578), and their interactions with APOE might increase the risk of LOAD [134].

Granados et al. first used a multidimensional dimensionality reduction (MDR) algorithm to perform epistasis analysis on 12 AD-related SNPs. The dataset used was composed of 196 AD cases and 92 controls [135]. Zieselman et al. also recognized the value of MDR, and they used a quantitative multifactor dimensionality reduction (QMDR) method to analyze the SNP-SNP interactions on the GWAS LOAD dataset from ADNI. They found statistically significant synergistic interactions between several SNPs, but the results were not repeated in another independent dataset [136]. To address the combinatorial explosion problem of large-scale GWAS datasets, Moore et al. introduced a method that combined expert knowledge and MDR methods to examine high-order gene-gene interactions. Expert knowledge from databases (such as gene ontology) or literature sources (such as PubMed) was used to filter gene datasets before the analysis. They applied this method to the GWAS dataset from ADNI and identified a set of interacting genes related to AD [137].

Another machine learning algorithm, iterative sure independence screening (SIS), can analyze very large datasets with more predictors than observations. An interaction analysis was performed by Hibar et al., which screened all possible SNP-SNP interactions that affected regional brain volumes from 534,033 SNPs in a GWAS dataset from ADNI. They found a significant SNP-SNP interaction between rs1345203 (probably related to histone acetylation) and rs1213205 (probably related to DNase I cleavage), which could explain 1.9% of the changes in the temporal lobe volume [138].

There are also many studies using machine learning methods to analyze transcript interaction networks. In an earlier study, Armananzas et al. used ensemble Bayesian network classifiers to build transcript interaction networks based on transcript profiling from entorhinal cortex and dentate gyrus samples in six AD and six control cases in 2012. Studies have found that some key transcripts in the network, such as S100A10, RPS3A, MED8, may have an important significance for the pathogenesis of AD [139]. The combinatorial optimization-based machine learning algorithm proposed by Ponzoni et al. was used to analyze the functional interconnection of two gene expression datasets in AD and control brain tissue. This approach provided both a global view of interconnections between different functional blocks and a specific molecular network of interest. The previously unreported AD-related pathways obtained by this method included the citrate cycle, pyruvate metabolism, MAPK signaling, peroxisome, VEGF signaling, focal adhesion, aldosterone-regulated sodium reabsorption, carbohydrate digestion and absorption [140].

Zafeiris et al. designed an integrated artificial neural network (ANN) pipeline for biomarker discovery and verification in AD. By analyzing a gene expression microarray dataset (E-GEOD-48350) consisting of no less than 80 cases and at least four brain region samples, they generated a large and complex interaction dataset consisting of 500 gene probes and 1,000 predicted interactions, which could be used as a reference system to further examine genes of interest. Driver analysis produced a list of the most influential and most influenced genes that may be the source of imbalances in the metabolic system and therefore the most likely driver and treatment target for the disease [141]. Similar to the study by Zafeiris et al., Park et al. proposed a random forest-based algorithm to classify important gene-gene interactions. They tested the trained algorithm on an AD gene expression dataset (GSE15222). As a result, 3,366 AD-associated gene-gene interactions were identified, and functional enrichment analysis showed that several AD-related pathways were significantly enriched [142].

Maj et al. took a different approach to study potential biological associations in different tissues with AD. They first applied a tissue-specific gene expression prediction model to predict the gene expression profiles of 42 nongender-specific tissues based on the genotypes of 808 samples from GWAS datasets of ADNI, which included controls, mild cognitive impairment subjects and AD patients. Then, the association between the AD cognitive decline and predicted tissue-specific gene expression was analyzed by different supervised and unsupervised machine learning methods. The advantage of using predicted transcriptome data is that it only reflects the role of genetic components and avoids environmental influences. Since epistatic interactions play a major role in the regulation of biochemical pathways, this study focused only on the analysis of regulatory networks, not univariate analyses. The results suggested that the inflammatory and regulatory processes in gut-brain-related tissues had a potential effect on the cognitive decline [143].

Machine learning has also been used in the study of metabolic pathways. Coppede et al. were skeptical about the impairments in folate metabolism potentially being a risk for AD and used ANN to analyze 30 genetic and biochemical variables related to folate metabolism on a dataset that included 40 LOAD cases and 40 matching controls. Upon analyzing the results, they created a semantic connection map that could show complex biological associations between variables to differentiate AD cases from controls. The study found that certain variables (such as the *TYMS* and *DNMT3B* genotypes) may play an important role when considering the interaction of multiple variables in this pathway [144]. From the above 13 studies, it can be concluded that machine learning has significant advantages in analyzing and mapping complex networks of genetic and metabolic interactions.

## Genetic Analysis Based on a Knowledge base

Most studies that used machine learning to decipher the pathogenesis of AD analyzed genetic or other medical information (such as brain imaging) from various original AD databases. However, there are few studies looking for alternative methods to help this development. These studies used AI technology to identify genes associated with AD risk by analyzing an established biological knowledge base.

Jamal et al. tried to find AD susceptible genes by employing eleven machine learning algorithms to analyze several open-source knowledge bases. The integrated topological properties of the AD-related genes were extracted from the protein-protein interaction networks (OPID, STRING, MINT, BIND and InTAct databases), sequence features (UniProt database) and functional annotations (DAVID and two additional Swiss-Prot functional annotation terms). They also used molecular docking methods to screen interactions between known drugs for AD and newly acquired AD-related proteins [145]. In addition, Huang et al. used an SVM method to integrate the information from an AD gene knowledge base (AlzGene) and the brain-specific gene network data from GIANT, and then they analyzed more than 20,000 genes in a catalog of human genes and genetic disorders (OMIM). The candidate gene list of 832 genes generated in this study might provide a comprehensive reference for AD gene research [146]. Text mining tools can facilitate the literature search process. Singhal et al. proposed a machine learning method that could automatically extract disease-gene-variation information from biomedical literature. They extracted the above information about ten important diseases, including AD, from all PubMed abstracts. After a comparison study with the UniProt knowledge base, the author believed that the method has practical value [147]. From this, we know that the integrated analysis of the knowledge base can provide some important research clues.

## Perspective for the Future

The computing power and capabilities of developing technology increase exponentially every year. These new technologies have enabled the analysis of complex biological processes and diseases with extraordinary size and numerous dimensions. Especially for complex diseases, including AD, analysis in a single or few dimensions prevents us from capturing the exact causes and factors associated with these diseases. Thus, efficient but complicated methods must be employed to combine multiple data types to pinpoint specific factors of a disease [16]. To the best of our knowledge, research papers on the genetics of AD using AI methods have only appeared in the last ten years, and many of them have focused on the exploration of research methods. In recent years, with the continuous increase in available public databases and the improvement of computer capabilities, research papers in this area have gradually increased. However, in general, the databases used for AI research are relatively limited, and few studies have applied a comprehensive analysis at multiple levels of genes, proteins, metabolism, and environmental factors. The conclusions of most studies are only to provide a reference for further research. Few studies have carried out a biological verification of the findings or proposed a verification scheme; in addition, few studies have proposed a theoretical framework for the pathogenesis of AD based on the results obtained [30, 58]. Regarding the use of AI technology to study the genetic factors of AD and the pathogenesis of AD, current challenges, possible solutions and future development directions are discussed below.

1 High quality, comprehensive, large sample size, and data sharing resources: the quality of the original data resources is the basic condition for obtaining correct results; comprehensive, large-sample data resources can improve researchers' ability to spot weak factors; in addition, shared data resources can provide opportunities for more research teams to participate in AD research. The ANDI database is a very successful example of this. Other large shareable databases have been listed above and include ADGC, ADSP, EMBL-EBI, GAAIN, NCBI, NIAGADS, etc. However, the majority of these data come from only developed countries and do not include major populations in developing countries. It is believed that future international cooperation will also promote the improvement and development of these shared databases [98]. For genome and gene expression databases, WGS and RNA-Seq based on next-generation sequencing can detect nonpredesigned sequences; thus, these have more advantages than microarray-based databases. We believe that such databases will play a more important role in the future. The organism as a whole is a complex system of genes, proteins, cells, individuals and environmental factors (including various physiological and biochemical conditions of the internal and external environments, including living conditions, lifestyle, social psychology, etc.) at different levels. Establishing and improving the dynamic database of the corresponding genome, epigenome, transcriptome, proteome, metabolome, microbiome and other internal and external environmental factors are the future development directions. Currently, the ability to collect data from internal and external environmental factors is still very limited. It is believed that with the development of nanotechnology [148], wearable devices [149], the Internet of things [150], smartphone applications [151], and other technologies [152], these dynamic databases will also be established. In addition, knowledge bases, including AlzGene and UniProt knowledge base, also play a vital role.
2 Multilevel system biological analysis strategy: AD is the result of the complex interactions between genes and the environment. Therefore, the study of its pathogenesis needs to include the interaction between the genome and environmental factors, as well as the epigenome, transcriptome, proteome, metabolome, and microbiome, in related cells and tissues. A section in this article specifically discusses the study of genes and gene interactions by AI technology. Indeed, these studies have also found many meaningful interactions for the pathogenesis of AD. Systems biology is an interdisciplinary research area that uses holistic methods to analyze complex interactions in biological systems through mathematical models [153]. We believe that this analysis strategy of systems biology combining various aspects of information will continue to progress with incredible advances in computing power, new AI algorithms and availability of data. Ultimately, a comprehensive AI analysis system can be established, which will be of great significance for a thorough understanding of the pathogenesis of complex diseases such as AD [16, 154].
3 Theoretical summary and verification of AI analysis results: Conclusions from most of the studies presented in this article only indicate that certain genes or pathways may be related to the pathogenesis of AD, and these only provide a reference for further research. Can the study of the pathogenesis of AD using AI technology only reach this stage? Of course not. The goal of theoretical research is to be able to propose a verifiable model of the research object. For simple objects, such as the motion of objects, this theoretical model can be expressed using very simple mathematical formulas, but for complex diseases, such as AD, due to the interaction of many factors, it can be speculated that this theoretical model must be a complex model built on a computer program [155]. Can the establishment of such a theoretical model only depend on the creativity of human thinking? Can AI help us build these complex models? We have reason to believe that AI technology can help. In fact, although computational creativity is still in its infancy, it is also one of the important branches of the rapid development of AI. It is committed to making computers have the ability to play independent creators or co-creators. Its application researches include literature and art creation (such as stories, poetry, paintings, musical works, games), problem solving, and system design, etc. [156, 157]. Some form of machine learning has been used in almost all the studies presented in this article. Although this method has indeed reached a certain level of practicality, machine learning is only a small part of AI technology. We boldly speculate that a more comprehensive AI technology including computational creativity and machine learning can not only help us make accurate diagnosis and prediction, but also help us analyze the research results, propose new hypotheses or theoretical models, design feasible verification schemes, and new intervention protocols. AI technology will bring humanity into a new era [56, 71, 158].

## Concluding Remarks

Most cases of AD are the result of a complex interaction of multiple genes and environmental factors, and traditional genetic analysis methods were successful in discovering many of the significant genes and factors for the pathogenesis of AD. In recent years, with the development of large databases such as GWAS, gene expression array, WGS, and RNA-Seq, the analysis and exploration of data by conventional statistical methods have shown certain limitations. AI technology (machine learning algorithms) has been applied to the analysis of genetic variations, gene expression profiles and gene-gene interactions of AD in the past 10 years and has produced some meaningful results. Although it is still at a relatively preliminary stage, we believe that with the continuous improvement of high-quality, comprehensive, large sample size, data sharing resources, applying multilevel system biological analysis strategies, and incredible advances in computing power, a comprehensive analysis system can eventually be established and help to fully understand the pathogenesis of AD. In the future, computational creativity may play a role in building and verifying a theory model and designing new intervention protocols for AD.
